# Multilayer Electrospun-Aligned Fibroin/Gelatin Implant for Annulus Fibrosus Repair: An In Vitro and In Vivo Evaluation

**DOI:** 10.3390/biomedicines10092107

**Published:** 2022-08-29

**Authors:** Ming-Hsiao Hu, Kai-Chiang Yang, Chih-Wei Chen, Po-Han Chu, Yun-Liang Chang, Yuan-Hui Sun, Feng-Huei Lin, Shu-Hua Yang

**Affiliations:** 1 Department of Orthopedics, National Taiwan University Hospital, National Taiwan University College of Medicine, Taipei 100225, Taiwan; 2 Department of Dental Technology, College of Oral Medicine, Taipei Medical University, Taipei 11031, Taiwan; 3 Department of Biomedical Engineering, National Taiwan University, Taipei 106216, Taiwan

**Keywords:** annulus fibrosis, electrospinning, fibroin, intervertebral disc, multilayer scaffold

## Abstract

Annulus fibrosus (AF) damage is proven to prompt intervertebral disc (IVD) degeneration, and unrepaired AF lesions after surgical discectomy may boost herniation of the nucleus pulposus (NP) which may lead to further compression of neural structures. Moreover, vascular and neural ingrowth may occur within the defect which is known as a possible reason for discogenic pain. Due to a limited healing capacity, an effective strategy to repair and close the AF defect is necessary. In this study, using electrospinning technology, two nature polymers, silk fibroin and gelatin, were linked to imitate the unique lamellae structure of native AF. Our findings revealed that a multilayer electrospun-aligned fibroin/gelatin scaffold with mechanical and morphological properties mimicking those of native AF lamellae have been developed. The average diameter of the nanofiber is 162.9 ± 38.8 nm. The young’s modulus is around 6.70 MPa with an ultimate tensile strength of around 1.81 MP along preferred orientation. The in vitro test confirmed its biocompatibility and ability to maintain cell viability and colonization. Using a porcine model, we demonstrated that the multilayer-aligned scaffold offered a crucial microenvironment to induce collagen fibrous tissue production within native AF defect. In the implant-repaired AF, H&E staining showed homogeneous fibroblast-like cell infiltration at the repaired defect with very little vascular ingrowth, which was confirmed by magnetic resonance imaging findings. Picrosirius red staining and immunohistochemical staining against type I collagen revealed positively stained fibrous tissue in an aligned pattern within the implant-integrated site. Relative to the intact control group, the disc height index of the serial X-ray decreased significantly in both the injury control and implant group at 4 weeks and 8 weeks (*p* < 0.05) which indicated this scaffold may not reverse the degenerative process. However, the results of the discography showed that the effectiveness of annulus repair of the implant group is much superior to that of the untreated group. The scaffold, composed with nature fibroin/gelatin polymers, could potentially enhance AF healing that could prevent IVD recurrent herniation, as well as neural and neovascular ingrowth after discectomy surgeries.

## 1. Introduction

The intervertebral discs (IVDs) comprise a hydrated and gelatinous nucleus pulposus (NP) core, surrounding the multilaminar annulus fibrosus (AF), and cartilaginous endplates [1,2]. The extracellular matrix (ECM) of NP contains water, proteoglycans, and collagen that provide the bearing capacity for the IVD to resist the mechanical load. The pathophysiological evidence demonstrates that the IVD degeneration initiates from the NP which lose collagens and proteoplycans with age. As the NP loses proteoglycans and becomes dehydrated, it would transfer greater loading to the AF as the intradiscal hydrostatic pressure decreases. Excessive loading to the AF would lead to fissures or tears in the AF. Discogenic pain, a common cause of lower back pain, may result from neovascularization and neoinnervation which infiltrate the IVD through peripheral AF [3]. The NP materials may herniate through the lesions of AF, leading to the so-called IVD herniation, which would compress the dura sac or spinal nerve roots and cause the sufferings of the patients [4,5]. When clinical symptoms are not responded to by nonsurgical managements such as steroid injection, surgery is required to remove extruded NP fragments in order to relieve symptoms. The most common surgical procedure is partial discectomy. However, current clinical practice does not seal the AF defects caused by surgical procedures for removal of disc fragments [6,7]. The untreated defects in the AF have been shown with limited healing capacity and are potential sites for recurrent disc herniation. Recurrent herniation following partial discectomy would lead to radicular pain and the recurrent rate was reported as 5–25% [8]. Repair of the AF lesions due to disc herniation or related surgical procedures would help to restore the containment of the NP and to provide an environment for IVD tissue regeneration.

Mechanical closure/suture and biologic repair are two major approaches which have been utilized in pre-clinical investigations of AF repair. Mechanical repair methods, including sutures and glues, attempted to close or block the AF lesions using biologically inert materials [9,10,11,12]. However, the current result of the in *ex* and in vivo ovine and porcine study showed that neither simple suturing [13] nor more complex suture systems, such as Dines knot [12] or modified purse-string sutures [14,15], could significantly enhance tissue integration, long term healing and regeneration, or restore native biomechanical function to the spine. Xclose^®^ (Anulex Technologies Inc., Minnetonka, MN, USA), an early commercial product to close the rupture site in the AF, was designed to make an X-shaped stitch using braided polyester bands. Apparently, it was discontinued since it has failed to neither improve AF healing nor withstand high mechanical loads [16,17]. Barricaid™ (Intrinsic Therapeutics, Woburn, MA, USA), a metallic-base implant and woven polyester component, was recently introduced as an AF closure device [18]. However, Barricaid™ was designed to fix to the adjacent vertebral body using a titanium bone anchor which draws the concern of initiating or accelerating IVD degeneration [19].

Biologic repair of AF consists of biomaterials attempting to imitate the native fibrous structure of AF with or without AF or stem cells [2]. Some natural and synthetic implants for AF repair have been investigated in vivo such as a small intestinal submucosa implant [20], a synthetic poly(lactic-co-glycolic acid) sponge [21], and a poly(trimethylene carbonate) combined with an elastic polyurethane membrane [22]. Cell migration and collagen formation have been found to appear within those implants. Furthermore, numerous studies have shown that the induction of AF cells and ECM deposition could be guided by the scaffold topography [23,24]. Taken together, these results emphasize that a 3D scaffold mimicking the native AF structures can guide cell migration, proliferation, and ECM deposition. They can help the implants fully integrate with the surrounding tissue, resulting in a solid AF repair.

The AF comprises a unique and complex structure of concentric annular lamellae. Each lamella consists of mostly elongated fibroblast-like cells and they produce mainly type I collagen. The lamellae are aligned and parallel to each other that are oriented at approximately 30° to the transverse plane, varying to −30° relative to the adjacent lamella. The thickness of the lamellae in the AF varies in the radial direction and is approximately 130 μm for the outer AF [25]. Using a variety of scaffolds, many studies have figured that 3D printing and electrospinning technologies are particularly suitable for developing highly organized micro- or nano-fibrous AF implants [26,27,28,29]. Polycaprolactone (PCL) has been widely recommended for AF repair and several in vivo studies described electrospun PCL implantation in a large animal model [30,31,32,33]. In a recent study, a cell-free biodegradable scaffold made of PCL with electrospun aligned microfibers, revealed high levels of aligned cell colonization and AF-like ECM deposition 4 weeks after lumbar discectomy in an ovine model [33].

In this study, two nature polymers are chosen as the implant material: silk fibroin and gelatin. Fibroin is a nature polymer with good biocompatibility, excellent mechanical properties, it is inexpensive, and a large amount of raw material is available from mature sericulture [34,35]. However, the hydrophobicity of fibroin makes it not a suitable environment for cell adhesion. In order to adjust the hydrophobicity, gelatin, a hydrophilic nature polymer with abundant tripeptide Arg-Gly-Asp (RGD) sequences, is mixed with fibroin to provide a better environment for cell attachment and proliferation [36,37]. Moreover, gelatin can swell after immersing in aqueous solution, thus we expect that the implant can absorb water and swell, blocking up the AF lesions after surgery. Electrospinning technology with a high-speed rotating roller is used [38,39] to design an aligned fibroin/gelatin scaffold that mimics native AF structure. The objective of this study is to demonstrate whether this cell free, aligned, electrospun fibroin/gelatin scaffold could induce organized fibrous tissue formation in vitro and in a porcine cervical spine model.

## 2. Materials and Methods

### 2.1. Isolation and Purification of Fibroin from Cocoon Silk

A Bombyx mori flat cocoon was purchased from a local sericulture farm. Gelatin Type A 175 Bloom and formic acid (98%) were purchased from Sigma-Aldrich (St. Louis, MO, USA). A Bombyx mori flat cocoon was degummed twice with 0.02 M Na_2_CO_3_ solution at 100 °C for 30 min and rinsed with warm distilled water to remove sericin. The degummed cocoon silk was put in the oven overnight to remove residual water and then dissolved in a solvent system of CaCl_2_/CH_3_CH_2_OH/H_2_O with a molar ratio of 1:2:8 at 80 °C for 30 min. The dissolved degummed silk was dialyzed against distilled water using the cellulose tubular membrane with the molecular weight cut off (MWCO) = 3500 for 72 h to remove salt and alcohol. The milky solution was dried by lyophilization, and the purified silk fibroin thus obtained was stored in a low humidity environment at room temperature for further studies [40,41].

### 2.2. The Preparation of Fibroin/Gelatin Multilayer Scaffold

The purified silk fibroin and gelatin were mixed with 95:5 in weight ratio and the polymer mixture was dissolved in 98 wt% formic acid and stirred overnight for electro-spinning. A 4 cm diameter running roller was used as a fiber collector to obtain a homogeneous fibrous membrane [42]. The parameter of the electro-spinning process used in the study was as follows: the distance between the needle tip and the collector was 8 cm and the needle input charge voltage was 17~18 kV. The flow rate of the syringe pump was 0.4–0.5 mL/h with a collector at an angular velocity of 3000 rpm. The single layer electrospun membrane was immersed in 95% alcohol to produce an insoluble membrane in phosphate buffered saline (PBS) [43]. The single layer electrospun membrane was immersed in the mixture of 5% gelatin solution and 0.1% microbial transglutaminase (mTG). Then, they were stacked layer by layer with the preferred orientation ±30° alternatively to mimic the native structure of the AF tissue. Finally, the scaffold was put in the 0.1% mTG for further crosslinking [44].

### 2.3. Characterization of Fibroin/Gelatin Electrospun Membrane and Fibroin/Gelatin Electrospun Scaffold

#### 2.3.1. Microstructure and Diameter of Electrospun Fibers and Multilayer Electrospun Implant

The microstructure of the cross section and surface morphology of the fibroin/gelatin electrospun membrane and multilayer electrospun implant were examined by back-scattering of electrons using a scanning electron microscopy (SEM) (JEOL, Tokyo, Japan) at an accelerating voltage of 10–15 kV. A thin platinum film was coated onto the SEM sample by sputtering physical vapor deposition. The average diameters of the electrospun fibers were analyzed using ImageJ^®^ (National Institutes of Health, Bethesda, MD, USA). The structure of annular lamellae of a native swine AF tissue was also observed using SEM.

#### 2.3.2. Tensile Strength Measurements

The tensile strength measurement of the fibroin/gelatin membrane was followed by the instruction of ASTM D882-10. In brief, the fibrous membranes were machined into a rectangle shape of 20 mm in length and 3 mm in width. The test sample was mounted on an MTS Test System at the elongation rate of 1 mm/min. Two perpendicular directions were examined due to the anisotropy. Several mechanical properties of electrospun membrane: Young’s modulus, ultimate tensile strength (UTS), elongation, and toughness were calculated based on the obtained stress–strain curve.

#### 2.3.3. Swelling Ratio

The fibroin/gelatin electrospun implant was immersed in PBS to allow for swelling in the aqueous solution. By measuring the weight at different times and according to formula 1, the swelling ratio of fibroin/gelatin electrospun scaffold could be calculated [45]:

(1) $$\mathrm{swelling}\;\mathrm{ratio}=\frac{W_{s}-W_{d}}{W_{d}}$$

$$W_{s}:\;\mathrm{weight}\;\mathrm{of}\;\mathrm{electrospun}\;\mathrm{scaffold}\;\mathrm{after}\;\mathrm{swell}\;\mathrm{in}\;\mathrm{aqueous}\;\mathrm{solution}$$

$$W_{d}:\;\mathrm{weight}\;\mathrm{of}\;\mathrm{electrospun}\;\mathrm{scaffold}\;\mathrm{in}\;\mathrm{alcohol}$$

### 2.4. In Vitro Analysis

#### 2.4.1. Cell Proliferation

L929 cell line, primary porcine AF, and NP cells (cultivated from porcine spine tissue purchased from PigModel Animal Technology Co., Ltd. (Miaoli, Taiwan (R.O.C.)) were separately seeded with a cell density of 10^4^ cells/well in 96 well for 24 h. The fibroin/gelatin electrospun membrane were immersed in culture medium with a concentration of 6 cm^2^/mL, and the extract was used to replace the medium in the 96 well (scaffold group). Extractions of zinc diethyldithiocarbamate (ZDEC) and aluminum oxide (Al_2_O_3_) were treated as the positive control and negative control, respectively. The regular culture medium was served as the control group. Cells were cultured in the extractions for 1 day, and the extractions were replaced as regular media containing 10% with WST-1 (Takara, San Jose, CA, USA) reagent and cultured for an additional 2 h at 37 °C. Cell viability was quantitatively assessed by spectrophotometer readout at 450 nm, with a reference wavelength at 650 nm.

#### 2.4.2. Live and Dead Staining

Primary porcine AF and NP cells with a cell density of 10^4^/96 well were seeded on the electrospun membrane and incubated for 24 h. Then, the live/dead staining (Calcein AM/EthD-1, Invitrogen™ LIVE/DEAD™ Viability/Cytotoxicity Kit (Invitrogen, Paisley, UK)) was used to distinguish cell survival. The cell was seeded on the slide glass with culture medium and 2% Triton X-100 were used as the negative control and positive control.

#### 2.4.3. Cell Adhesion

Primary AF and NP cells were seeded on the electrospun membrane and incubated for 24 h and 72 h, and the cells on the membranes were then treated with 4% paraformaldehyde for chemical fixation. The critical point drying method was used to maintain the original structure of the cells. A thin platinum film was coated onto the sample by sputtering PVD before SEM observation.

### 2.5. In Vivo Analysis

#### 2.5.1. Ethical Aspects and Animals

Three Lanyu pigs (10 months old and weighing 33.8 ± 1.4 kg; purchased from Taitung Animal Propagation Station of the Livestock Research Institute, Taiwan. Animal housing location: PigModel Animal Technology Co., Ltd., Miaoli County, Taiwan) were operated in this study. The animals were maintained in accordance with the guidelines for the care and use of laboratory animals. Experimental protocols and surgical procedures were approved by the National Taiwan University Hospital College of Medicine Institutional Animal Care and Use Committee (IACUC) and IACUC of Pigmodel Animal Technology Co., Ltd. Five cervical IVDs (C2-C7) per pig was used for the experiments and the conditions were as follows: intact control (healthy group)—2 IVDs; injury control (unrepaired group)—1 IVD; and implant (repaired group)—2 IVDs.

**Figure 1:**
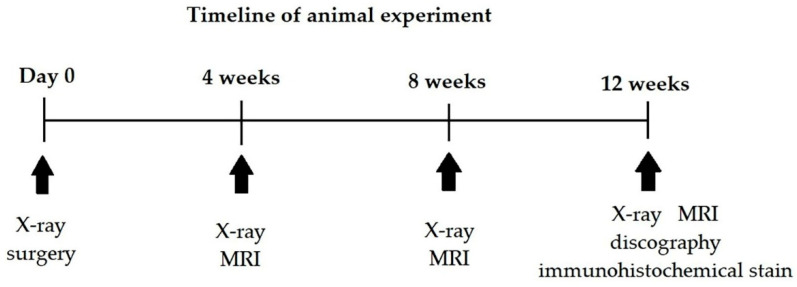
Timeline of animal study.

The timeline of the animal study is shown in Figure 1. The whole period of the in vivo study is 12 weeks. Several examinations were done to evaluate the effectiveness of AF repair at pre-determined intervals postoperatively.

#### 2.5.2. Surgery and Tissue Harvesting

Anesthesia was induced by intravenous injection of 3–5 mg/kg of Azeperonum (40 mg/mL; China Chemical & Pharmaceutical Co., Ltd., Taipei, Taiwan), 0.03–0.05 mg/kg of atropine (1 mg/mL), and 3–5 mg/kg Zoletil-50 (Vibac, Carros, France), and maintained by inhalation of 0.5–2% isoflurane. The animals were fixed in a supine position; the operating field was prepared from the submandibular area to the base of the neck. Under aseptic conditions, a longitudinal skin incision at the left side approach was performed. Then, the subcutaneous fascia was incised, followed by muscle dissection. The trachea, esophagus, and common carotid artery were protected and retracted and the desired intervertebral space was identified. All IVDs were assigned to the following groups randomly: either no defect induced (intact control group), a 3 × 5 mm box annulotomy was created in the AF without repair (injury control group), and a 3 × 5 mm box annulotomy was created in the AF with an implantation of the fibroin/gelatin electrospun implant into the AF defect (implant group). Postoperatively, all animals fully recovered from general anesthesia. The pigs were housed individually and fed with a controlled diet. All pigs were mobile the following day and no postoperative complications were observed. Cefazolin 15 mg/kg IM was given pre- and postoperatively for the next 7 days. Meloxicam 0.4 mg/kg IM was given as analgesic preoperatively and ketoprofen 1–3 mg/kg IM twice a day for 3 days and meloxicam 0.1–0.4 mg/kg per os twice a day for 5–7 days as analgesic postoperatively.

Twelve weeks after the surgery, all three pigs were sacrificed under general anesthesia via IV injection of overdose pentobarbital. One pig received discography before sacrifice and cervical spines were harvested in the other two pigs and immediately fixed with 10% paraformaldehyde.

#### 2.5.3. X-ray Imaging, Magnetic Resonance Imaging (MRI) and Discography

Using the same general anesthesia as described above, radiographs were captured preoperatively and at 4, 8, and 12 weeks after the index procedure. Anteroposterior and lateral radiography of the pig cervical spine were taken. The IVD height was expressed as the disc height index (DHI) based on the method outlined by Mazuda [46]. The DHI was calculated by averaging disc height measured from the one fourth, middle, and three-fourths portion of the adjacent end plate width and dividing that by the average of adjacent vertebral body heights at the same points. This method has been validated for its validity and reliability previously [47,48].

Magnetic resonance imaging (MRI) was performed under general anesthesia as described above, animals received MRI examination 4, 8, and 12 weeks postoperatively using the 3T MRI system (Achieva X 3.0, Philips, Amsterdam, The Netherlands), using a standard phase array spinal coil, to acquire T2-weighted images in sagittal plane (TR = 800.0 ms; TE = 22.0 ms; slice thickness = 2.0 mm) in National Applied Research Laboratories and Taiwan Instrument Research Institute.

Discography is a clinical method to examine the integrity of annulus fibrosus. Under anesthesia, the study IVDs were explored through a previous surgical scar. Contrast medium (Iohexol, Omnipaque, GE Healthcare, Oslo, Norway) was injected into the IVDs by 25G needle under X-ray guidance. Judging from whether the contrast medium is well contained or leaks outside via an AF defect, one can evaluate the effectiveness of AF repair. The Adams grading system, which is proven consistently reproducible amongst observers with differing levels of experience [49], is used for discogram morphology.

#### 2.5.4. Histological and Immunohistochemical Staining

Porcine cervical discs were fixed in 10% neutral buffered formaldehyde solution for 2 days, and subsequently immersed in a decalcification solution (0.5 M aluminium chloride, 8.5% HCl, and 5% formic acid) for 3 days of decalcification. Following decalcification, samples were prepared for histological inspection and the section specimen was then stained with hematoxylin and eosin (H&E) and picrosirius red.

For immunostaining for type I collagen, the sections were immersed in a solution of methanol with 3% H_2_O_2_ for 10 min to quench endogenous peroxidase activity and then incubated in a citrate buffer (pH 6.0) antigen retrieval solutions for 30 min at 95 °C. Following overnight incubation of the slides in type I collagen antibody (5 μg/mL overnight at 4 °C; ab34710, Abcam, Cambridge, UK), the sections were labeled with streptavidin-biotin. The presence of antigens was detected using 3,3′-diaminobenzidine tetrahydrochloride substrate (Mouse and Rabbit Specific HRP/DAB Detection IHC kit (ab64264, Abcam, Cambridge, UK)). Sections were further counterstained with hematoxylin.

### 2.6. Statistical Analysis

All the results were expressed as average ± SD. Statistical analysis was performed using a Student’s t test. In addition, one-way ANOVA was utilized to evaluate the *p* value. The differences were considered as significant at *p* < 0.05.

## 3. Results

### 3.1. Fibroin/Gelatin Scaffold Characterization

**Figure 2:**
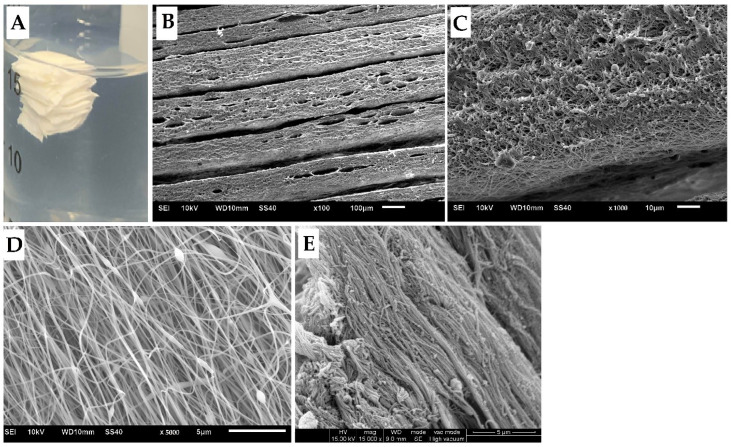
The appearance and SEM images. (**A**) The gross appearance of multilayer electrospun implant. (**B**) SEM image of multilayer electrospun implant from the side, 100×. (**C**) SEM image of multilayer electrospun implant from the side, 1000×. (**D**) SEM image of electrospun membrane from the top, 5000×. Average fiber diameter is 245.3 ± 50.7 nm (*n* = 100). (**E**) SEM image of native AF tissue, 5000×. Average fiber diameter is 162.9 ± 38.8 nm (*n* = 100).

The appearance and SEM image of the fibroin/gelatin electrospun membrane and multilayer electrospun implant are shown in Figure 2. A scaffold with uniform distributed nanofiber with a diameter around 200–300 nm and preferred orientation is produced, which mimics the size of a native AF tissue fiber but is a little bit thicker (average diameter is 162.9 ± 38.8 nm). From the side view of an electrospun scaffold, one can observe the layer-by-layer structure at low magnification and porosity between nanofibers at high magnification.

**Figure 3:**
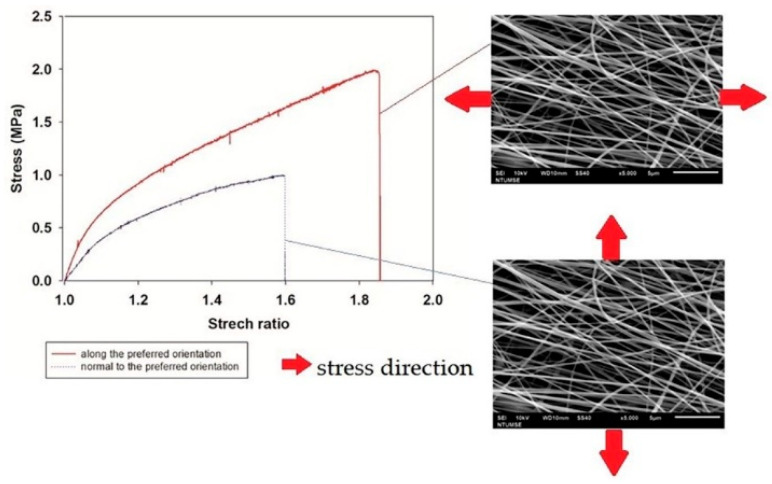
Stress–strain curve of fibroin/gelatin electrospun membrane. Red curve is the result when stress is along the preferred orientation. Blue curve is the result when stress is perpendicular to the preferred orientation.

The stress–strain curve of fibroin/gelatin electrospun membrane is shown in Figure 3. Two stress directions were applied to the electrospun membrane due to the anisotropy. The young’s modulus is around 6.70 MPa, ultimate tensile strength (UTS) is around 1.81 Mpa, elongation is around 90%, and the toughness is around 1.1 MJ/m^3^ for stress along preferred orientation. Although the young’s modulus is around 3.18 MPa, UTS is around 0.96 MPa, elongation is around 60%, and the toughness is around 0.3 MJ/m^3^ for stress perpendicular to the preferred orientation. It is observed that stress along the preferred orientation exhibited much better mechanical properties. Since the multilayer implant was produced by stacking these membranes layer-by-layer with ±30° alternatively, one can infer that the mechanical properties of the fibroin/gelatin multilayer scaffold would fall in between.

The mechanical properties of the dorsal site of native AF tissue were listed in Table 1 in comparison with the fibroin/gelatin electrospun membrane since dorsal sites are the most possible site for herniation of IVD. It is revealed that all the mechanical properties are similar or superior to native AF tissue, hence the multilayer scaffold may provide adequate mechanical support for AF repair.

**Figure 4:**
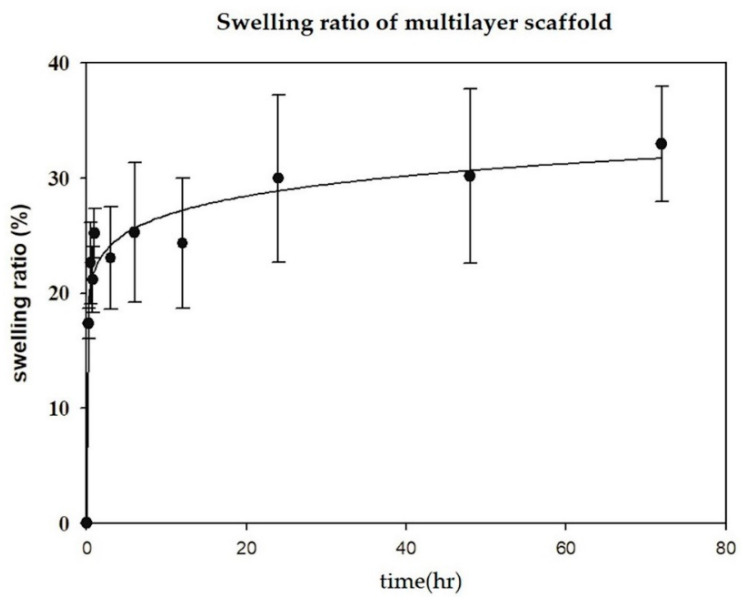
Swelling property of fibroin/gelatin electrospun implant. (*n* = 5).

The result of the swelling ratio of the fibroin/gelatin electrospun implant is shown in Figure 4. It revealed that the multilayer electrospun implant would swell by 20–30% quickly within 3 h in aqueous solution, and the swelling ratio increased slowly thereafter. The property would provide fast scaffold adherence at AF defect after surgery.

### 3.2. In Vitro Analysis

#### 3.2.1. Scaffold Biocompatibility

**Figure 5:**
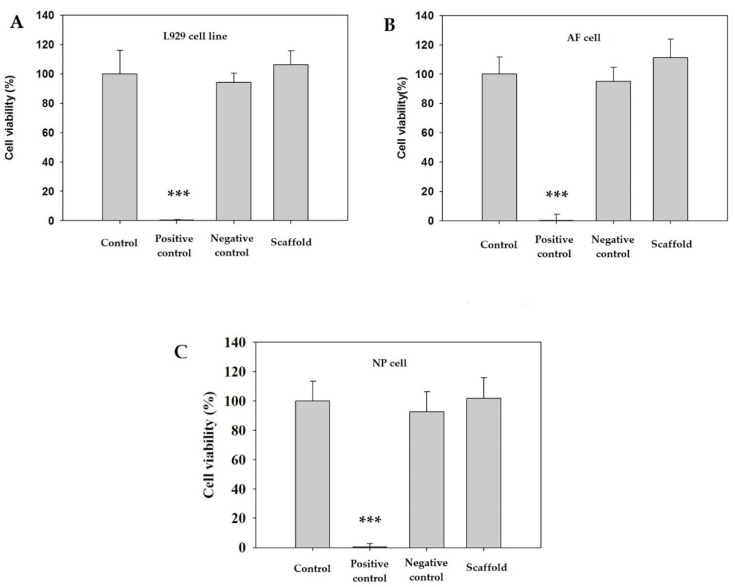
The results of WST-1 assay ratio of different cells with fibroin/gelatin electrospun membrane: (**A**) L929 cell line; (**B**) primary AF cell; (**C**) primary NP cell. Extraction of zinc diethyldithiocarbamate (ZDEC) and aluminum oxide was treated as positive control and negative control. ***: *p* < 0.001 by *t*-test (*n* = 6).

The biocompatibility of fibroin/gelatin electrospun membrane was determined by WST-1 assay. The results are shown in Figure 5. It revealed that the fibroin/gelatin electrospun membrane had great biocompatibility in the L929 cell, primary porcine AF, and NP cells. No significant differences were noticed among control, negative, and scaffold groups in L929, AF, and NP cells.

#### 3.2.2. Fluorescence-Based Live/Dead Staining

**Figure 6:**
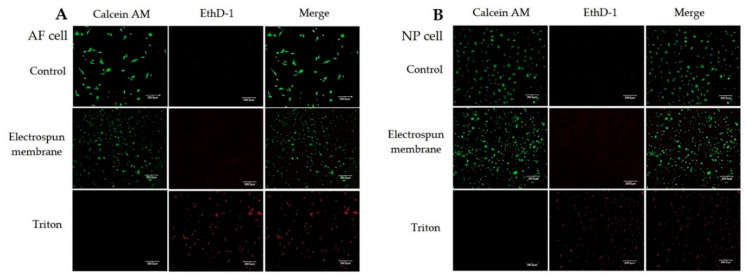
The results of live/dead staining: (**A**) primary AF cells, and (**B**) primary NP cells, cultured on the slide glass (control group), fibroin/gelatin electrospun membrane, or treated with Triton X-100.

The results of the fluorescence-based live/dead staining of the fibroin/gelatin electrospun membrane are shown in Figure 6. Both porcine AF and NP cells could adhere on the electrospun membrane without obvious cell death. On the contrary, cells treated with triton had many dead cells. The results supported that the fibroin/gelatin electrospun membrane had great biocompatibility in both AF and NP cells, as revealed in WST-1 assay.

#### 3.2.3. Scaffold Surface Adhesion and Cell Proliferation

**Figure 7:**
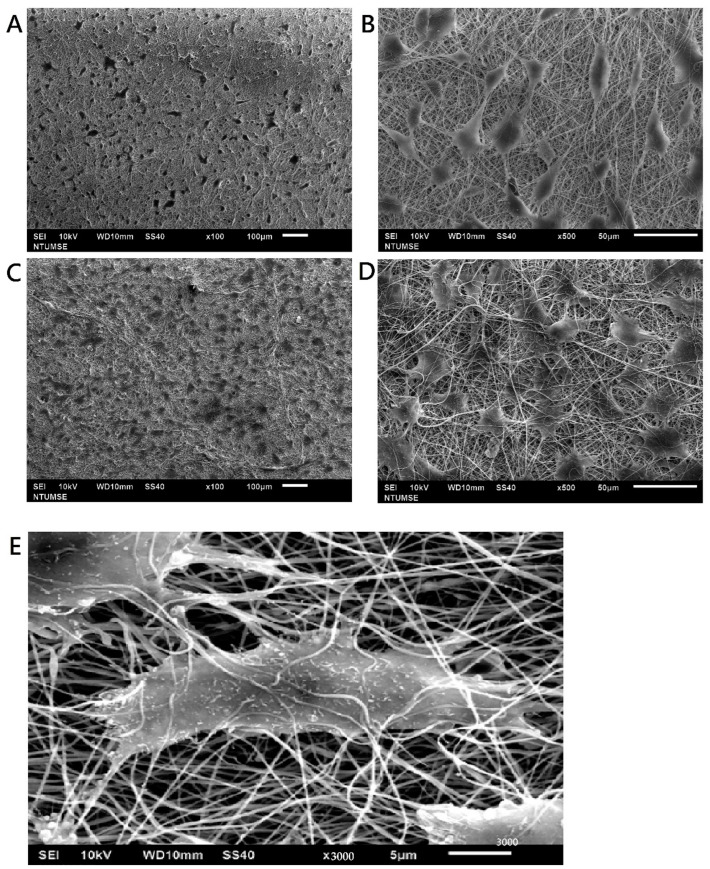
SEM images of primary porcine AF cell morphology on fibroin/gelatin electrospun membrane: (**A**) 24 h, 100×; (**B**) 24 h, 500×; (**C**) 72 h, 100×; (**D**) 72 h, 500×; (**E**) 72 h, 3000×.

**Figure 8:**
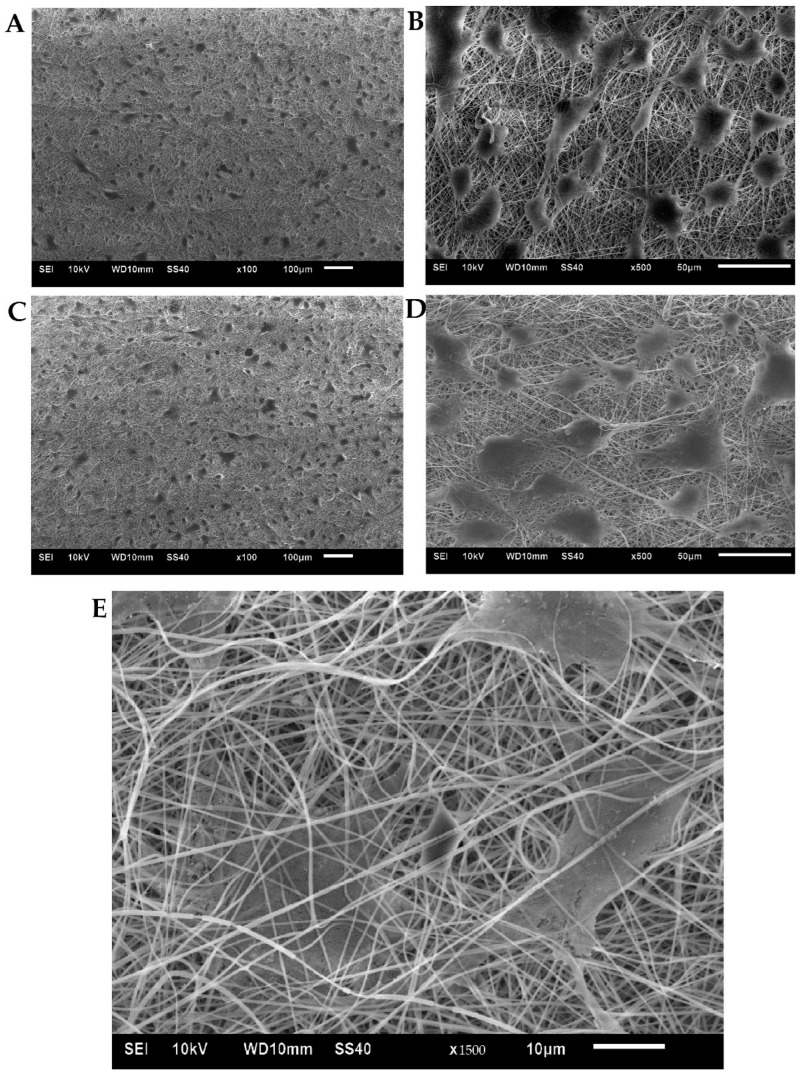
SEM images of primary porcine NP cell morphology on fibroin/gelatin electrospun membrane: (**A**) 24 h, 100×; (**B**) 24 h, 500×; (**C**) 72 h, 100×; (**D**) 72 h, 500×; (**E**) 72 h, 1500×.

AF (Figure 7) and NP cells (Figure 8) colonizing the scaffold were identified by SEM. It was observed that cell adhesion would align with fiber orientation, which implied that fibers with preferred orientation could guide the growth direction of AF and NP cells. Most cells adhered well on the fibroin/gelatin electrospun membrane after 24 h and integrated into the pores within 72 h. Moreover, cells may migrate into the pores between electrospun fibers which indicates that the cell could migrate to the fibroin/gelatin electrospun implant to facilitate AF defect repair and tissue healing.

### 3.3. In Vivo Implantation

#### 3.3.1. X-ray Imaging, MRI and Discography

Five cervical IVDs (C2-C7) per pig were used for the experiments, including two intact controls (healthy group), one injury control (unrepaired group), and two implants (repaired group). As shown in Figure 1, for each study animal, a pre-op X-ray was taken where a serial X-ray as well as an MRI was done at 4, 8, and 12 weeks after the index surgery. One pig received discography before sacrifice.

**Figure 9:**
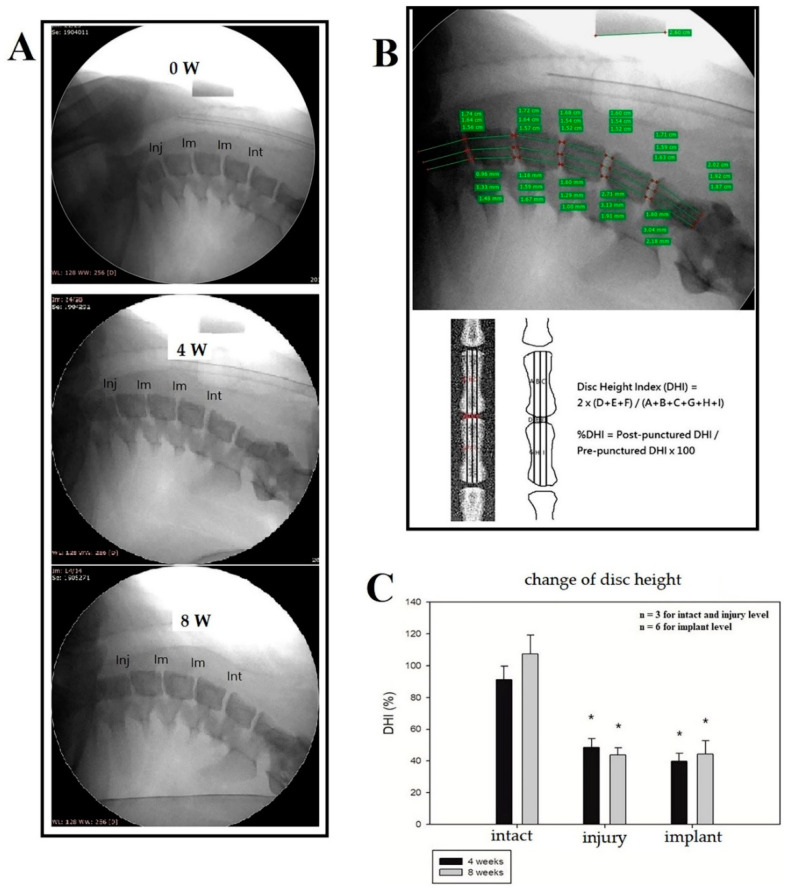
(**A**) Representative radiographs of the porcine cervical discs before, 4 weeks, and 8 weeks after the surgery (Int: intact; Im: implant; Inj: injury). (**B**) Demonstration of disc height measurement using image J software and definition of disc height index. (**C**) % DHI decreased significantly in both injury control and implant group, compared to those in intact control group at 4 weeks and 8 weeks. * *p* < 0.05 by *t*-test (*n* = 3 for intact and injury group; *n* = 6 for implant group).

The representative radiographs of the porcine cervical discs before, 4 weeks, and 8 weeks after the surgery were shown, respectively, in Figure 9A. Disc height measurement was done using image J software and DHI was calculated. (Figure 9B) We found that %DHI decreased significantly in both the injury control and implant group, compared to those in the intact control group at 4 weeks and 8 weeks, but the difference between the injury and implant segment was not significant. (Figure 9C). The results indicated that progressive NP loss and degeneration occurs even after just annulotomy.

**Figure 10:**
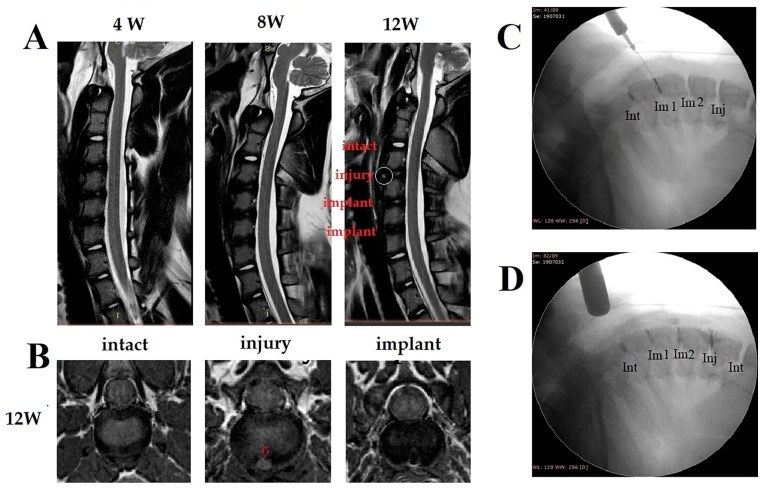
(**A**) T2-weighted MRI sagittal images at 4, 8, 12 weeks after surgery. The white circle indicates the formation of high intensity zone (HIZ) in injury group. (**B**) T2-weighted MRI axial images at 12 weeks after surgery. The red arrow indicates HIZ in injury group. HIZ was found within AF in injury control level at 12 weeks after the surgery, which indicated inflammation with possible peripheral vascular ingrowth. (**C**,**D**) shows the result of discography at 12 weeks after surgery. Type I (cotton ball appearance) in intact healthy disc (Int) and type III appearance (contained but irregular) in disc repaired with implant 1 (Im1) are shown in panel C. In injury group (Inj), after injecting contrast medium, immediate continuous leakage of contrast medium was shown which indicated failure of AF healing (**D**).

The T2-weighted MR images are shown in Figure 10A. It was observed that the NP degenerated in both the injury control group and implant group compared to that of the control group. The T2-weighted MRI at 12 weeks showed a hyperintense foci that appeared in the AF annulotomy site of the injury control group, while a similar lesion was not found in all implant-repaired AF defects. This may be the high-intensity zone (HIZ), which is usually correlated to focal inflammation and neovascular ingrowth. HIZ is one such lumbar phenotype, which is reported as potential imaging biomarkers related to a symptomatic disc. These MRI findings showed that the implant fabricated in this study may serve as a barrier to block the ingrowth of a peripheral blood vessel.

The results of discography at 12 weeks postoperatively are shown in Figure 10C,D. The discogram showed type I (cotton ball appearance) in the intact healthy disc which meant no signs of NP degeneration. It indicated that there was no damage in the AF site in the intact control group. In AF-repaired discs, one (implant 1) showed type III appearance (contained but irregular) which meant a degenerated disc with fissures and clefts in the inner nucleus. The other (implant 2) showed type IV appearance (fissured) which meant a degenerated disc with radial fissures leading to the outer edge of annulus. In the injury group, after injecting the contrast medium, immediate continuous leakage of the contrast medium was shown which indicated failure of AF healing. These findings verified that spontaneous healing of AF defect is not easy after surgical annulotomy or discectomy. The results of discography showed that the effectiveness of annulus repair of the implant group is much superior to that of the untreated group.

#### 3.3.2. Histological and Immunohistochemical Staining Analysis

**Figure 11:**
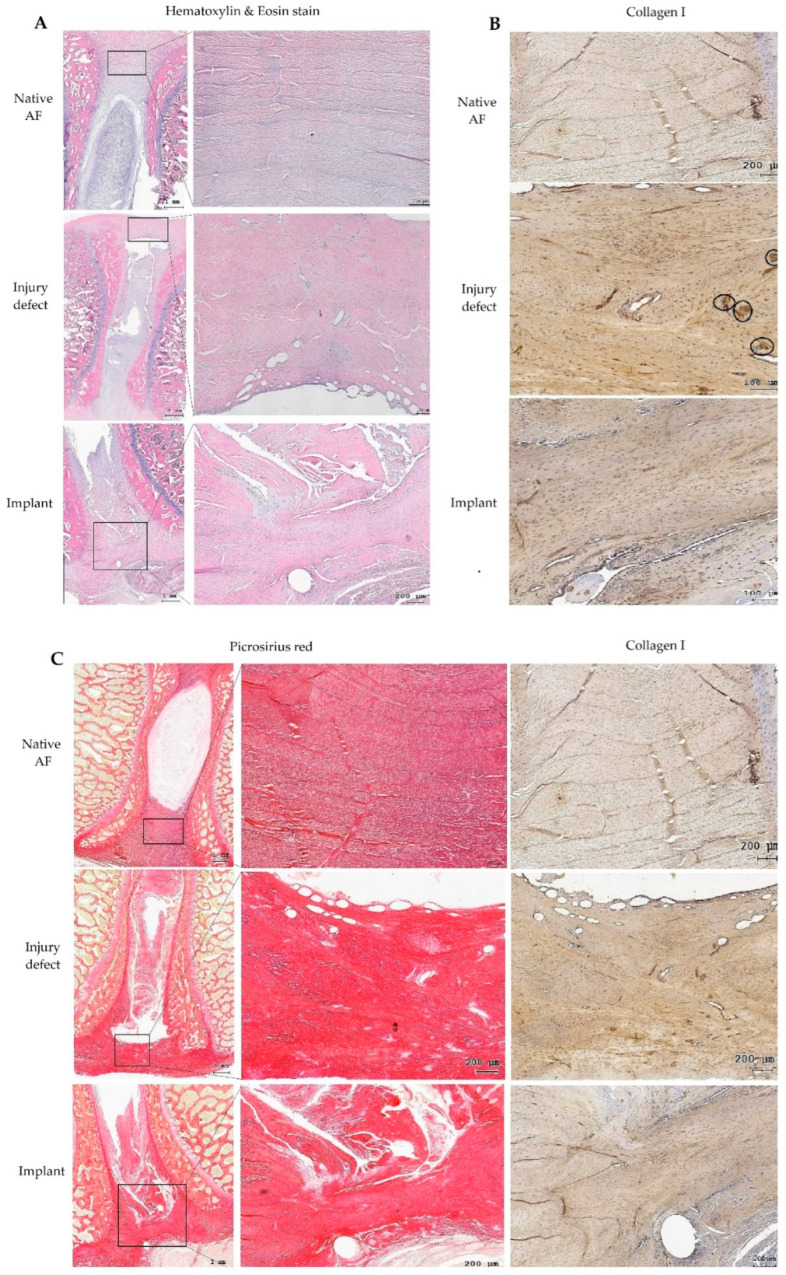
In vivo study with explanted intervertebral disc at 12 weeks after the surgery. (**A**) H&E staining in control group demonstrated a native AF with regular and intact inner AF lamellae. In injury group, empty defect without implant showed irregular fibrous reparative tissue with highly cellularized with fibroblast-like cells. In the implant-repaired AF, the multilayer fibroin/gelatin scaffolds were well healed within the outer AF defects without any sign of dislodgement. (**B**,**C**) Picrosirius red staining and type I collagen immunohistochemical staining revealed positively stained fibrous tissue in an aligned pattern within the implant-integrated site. In the injury defect group, this collagen-rich tissue was disorganized with no preferential orientation of the neosynthesized collagen fibers with numerous vascular ingrowths. (black circles, panel B).

Histological staining of decalcified IVDs with H&E staining in the control group demonstrated a native AF with regular and intact inner AF lamellae. In the injury group, the empty defect without implant showed irregular fibrous reparative tissue at 12 weeks. This tissue was highly cellularized with fibroblast-like cells displaying elongated nuclei. In the implant-repaired AF, the multilayer fibroin/gelatin scaffolds were almost resorbed at 12 weeks of implantation but were well healed within the outer AF defects without any sign of dislodgement. There was no sign of foreign body reaction around the implants and they were well integrated into the surrounding AF, with continuous collagen fibers. Homogeneous cell infiltration, most of them were fibroblast-like cells with elongated nuclei, was found at the repaired defect with very little vascular ingrowth (Figure 11A).

The observation of picrosirius red staining and immunohistochemical staining against type I collagen revealed positively stained fibrous tissue in an aligned pattern within the implant-integrated site. In the injury defect group, this collagen-rich tissue was disorganized with no preferential orientation of the neosynthesized collagen fibers with numerous vascular ingrowths at 12 weeks. (Figure 11B,C)

## 4. Discussion

AF damage is proven to prompt IVD degeneration and is widely accepted as one of the useful methods to induce IVD degeneration in animal models [33,50,51]. The results of the X-ray and MRI in this study also showed that progressive NP loss and degeneration occurred even after just annulotomy. In our previous study, it showed that we could induce a rapid degenerative process in rat caudal discs even by a needle puncture sized 21G or larger [48]. In this study, a 3 × 5 mm box annulotomy was created in the AF which was supposed to be a reason for the lack of statistical significance of DHI between the injury and implant groups. Unrepaired AF lesions may boost herniation of the NP which may lead to further compression of neural structures. Moreover, vascular and neural ingrowth may occur within the defect which is known as a possible reason of discogenic pain [3,52]. In the current study, the T2-weighted MRI at 12 weeks showed a hyperintense foci that appeared in the AF annulotomy site of the injury control group and immunohistochemical staining confirmed numerous vascular ingrowths in the AF defect of the injury group. These findings implied an inflammatory zone with neovascular ingrowth. An effective AF closure and repair is thought to be beneficial in minimizing IVD degeneration, preventing recurrent IVD herniation after surgical discectomy and prevention of neural and vascular ingrowth.

The AF is arranged in concentric annular lamellae consisting of packed aligned collagen fibers [25]. It is hypothesized that an ideal material which can achieve an effective biologic AF repair should approximate the ability of the native tissue in withstanding mechanical stress of the IVD and guide the migration of AF cells. In the present study, our findings indicated that the fibroin/gelatin multilayer electrospun implant with a fibroin-gelatin weight ratio of 95 to 5 could meet the requirement. In our preliminary results, the mechanical strength of the scaffold would be less than the native AF fibers with a higher percentage of gelatin. Then, gelatin is used as an adhesive to stack the membrane layer by layer with a specific angle alternatively, and its hydrophilic character improves the unfriendly environment for cell adhesion resulting from hydrophobicity of fibroin. The electrospun multilayer implant was analyzed by SEM to confirm that the microstructure mimics the native AF tissue with the fiber diameter being around 200–300 nm with preferred orientation. The result of the tensile test showed several important mechanical properties of the single layer electrospun membrane that are similar or superior to native AF tissue. The swelling capacity of the developed scaffold would provide better adherence to the surrounding AF tissues.

In the current study, the electrospun implant had great biocompatibility confirmed by WST-1 and cell survival staining. The SEM images show that both the AF cell and NP cell can adhere on the electrospun membrane and migrate into the pores of the electrospun fiber. Numerous cells were seen within each fibrotin layer of the aligned scaffold, indicating that the implant porosities were appropriate to induce cell infiltration and tissue ingrowth throughout each layer. According to the findings of histologic staining and DHI data, it suggested that NP degeneration occurred once the AF was injured, even when the defect was repaired using the current implant. However, based on the MRI findings of the in vivo study, it showed that the multilayer electrospun implant developed in this study may prevent the formation of a high intensity zone, suggesting that it can block the ingrowth of peripheral blood vessels which is associated with discogenic pain. The discography showed immediate continuous leakage of contrast medium in the injury group and it provided direct evidence that the effectiveness of the annulus repair of the implant group is much superior to that of the untreated (injury) group. It is thought that a contained environment would help to restore cell characteristics of the NP cells and to provide an environment for IVD tissue regeneration.

To the best of our knowledge, this in vivo study is the first to demonstrate a successful biological repair of AF defect without an immunologic reaction around the implant, using electrospun-aligned structures with pure nature polymers in a porcine spine model. Observation of picrosirius red staining and immunohistochemical staining of then type I collagen revealed positively stained fibrous tissue in an aligned pattern within the implant-integrated site. The effectiveness of annulus repair was confirmed by the results of the discography. There are still some limitations. First, the number of experimental animals is too small to achieve a statistic power. Second, the swelling capacity of the scaffold is 20–30% in aqueous solution, which is still inadequate for an immediate secure adherence to the surrounding AF tissue. Third, since we tested the implant in the porcine cervical spine, the resistance to mechanical loading in the human lumbar spine is still questionable.

## 5. Conclusions

In this study, a multilayer-aligned fibroin/gelatin scaffold with mechanical and morphological properties that mimicked those of native AF lamellae was successfully developed. The in vitro test confirmed its biocompatibility and ability to promote cell proliferation and colonization. Using a porcine model, it was demonstrated that the multilayer-aligned scaffold offered a crucial microenvironment to induce collagen fibrous tissue production within native AF defect. The scaffold, composed with nature polymers, could potentially enhance AF healing that could prevent IVD recurrent herniation, as well as neural and neovascular ingrowth after discectomy surgeries.

## Figures and Tables

**Table 1:** Comparison of mechanical properties of dorsal site of native tissue and fibroin/gelatin electrospun scaffold.

	Young’s Modulus (MPa)	UTS (MPa)	Elongation (%)	Toughness (MJ/m^3^)
native AF tissue (dorsal internal)	3.8	1.2	21	0.052
native AF tissue (dorsal external)	8.01	2.2	7.5	0.04
scaffold (along orientation) n=10	6.70 ± 2.59	1.81 ± 0.47	90.27 ± 18.36	1.103 ± 0.322
scaffold (normal to orientation) n=10	3.18 ± 1.22	0.96 ± 0.23	59.93 ± 9.88	0.311 ± 0.063

## Data Availability

The relevant data generated and (or) analyzed in the current study are available from the corresponding author upon reasonable request.
